# A Spectrum of Severe Familial Liver Disorders Associate with Telomerase Mutations

**DOI:** 10.1371/journal.pone.0007926

**Published:** 2009-11-20

**Authors:** Rodrigo T. Calado, Joshua A. Regal, David E. Kleiner, David S. Schrump, Nathan R. Peterson, Veronica Pons, Stephen J. Chanock, Peter M. Lansdorp, Neal S. Young

**Affiliations:** 1 Hematology Branch, National Heart, Lung, and Blood Institute, National Institutes of Health, Bethesda, Maryland, United States of America; 2 Laboratory of Pathology, National Cancer Institute, National Institutes of Health, Bethesda, Maryland, United States of America; 3 Thoracic Oncology Section, Surgery Branch, National Cancer Institute, National Institutes of Health, Bethesda, Maryland, United States of America; 4 Division of Cancer Epidemiology and Genetics, National Cancer Institute, National Institutes of Health, Bethesda, Maryland, United States of America; 5 Hematology Department, Hospital Vall d'Hebron, Barcelona, Spain; 6 British Columbia Cancer Agency and Department of Medicine, University of British Columbia, Vancouver, British Columbia, Canada; Washington University School of Medicine, United States of America

## Abstract

**Background:**

Telomerase is an enzyme specialized in maintaining telomere lengths in highly proliferative cells. Loss-of-function mutations cause critical telomere shortening and are associated with the bone marrow failure syndromes dyskeratosis congenita and aplastic anemia and with idiopathic pulmonary fibrosis. Here, we sought to determine the spectrum of clinical manifestations associated with telomerase loss-of-function mutations.

**Methodology/Principal Findings:**

Sixty-nine individuals from five unrelated families with a variety of hematologic, hepatic, and autoimmune disorders were screened for telomerase complex gene mutations; leukocyte telomere length was measured by flow fluorescence *in situ* hybridization in mutation carriers and some non-carriers; the effects of the identified mutations on telomerase activity were determined; and genetic and clinical data were correlated. In six generations of a large family, a loss-of-function mutation in the telomerase enzyme gene *TERT* associated with severe telomere shortening and a range of hematologic manifestations, from macrocytosis to acute myeloid leukemia, with severe liver diseases marked by fibrosis and inflammation, and one case of idiopathic pulmonary fibrosis but not with autoimmune disorders. Additionally, we identified four unrelated families in which loss-of-function *TERC* or *TERT* gene mutations tracked with marrow failure, pulmonary fibrosis, and a spectrum of liver disorders.

**Conclusions/Significance:**

These results indicate that heterozygous telomerase loss-of-function mutations associate with but are not determinant of a large spectrum of hematologic and liver abnormalities, with the latter sometimes occurring in the absence of marrow failure. Our findings, along with the link between pulmonary fibrosis and telomerase mutations, also suggest a common pathogenic mechanism for fibrotic diseases in which defective telomere repair plays important role.

## Introduction

Telomeres consist of tandem TTAGGG repeats and associated proteins located at the ends of chromosomes that serve to prevent recombination, end-to-end fusion, and activation of DNA damage responses [1]. Telomere attrition occurs with each cell division as a result of DNA polymerase's inability to replicate the extreme 3′-end of template strands [2], [3]. Progressive telomere shortening signals proliferation arrest and cellular senescence via p53, p21, and PMS2 [4]. In order to maintain proliferative capacity without compromising chromosome stability, embryonic and adult stem cells and certain somatic cells counter telomeric attrition by telomerase-catalyzed addition of TTAGGG repeats to the 3′ telomeric overhangs [5]. Constitutional loss-of-function telomerase mutations result in rapid telomere shortening and premature cellular senescence in proliferative somatic tissues [6].

Defective telomere repair has been causally associated with several human diseases. Genetic linkage analysis of the constitutional marrow failure syndrome dyskeratosis congenita led to the discovery of mutations in the genes *DKC1* (which encodes dyskerin) [7] and telomerase RNA component *(TERC)* [8]. We reported mutations in *TERC* [9] and telomerase reverse transcriptase (*TERT*) [10] to be risk factors for apparently acquired aplastic anemia, a marrow failure disease occurring in patients who lack the typical physical anomalies and family history of dyskeratosis congenita. Mutations in telomerase complex genes also appear in families with idiopathic pulmonary fibrosis [11]–[13], and pulmonary disease, esophageal stricture, malignancy, and liver disease also have been reported in twenty, seventeen, eight, and seven percent of dyskeratosis congenita patients, respectively [14].

Prior investigation of the clinical manifestations related to telomerase deficiency has been restricted to the screening of patient cohorts with specific diagnoses (bone marrow failure, pulmonary fibrosis). Using a different approach, we studied the family members of five unrelated patients with marrow failure and telomerase mutations; we genetically screened relatives who had a variety of hematologic, hepatic, and autoimmune disorders. We found that mutations were associated with a wide range of hematologic abnormalities, from macrocytosis to acute myeloid leukemia, and with severe liver diseases characterized by fibrosis, inflammation, and regeneration occurring independently of marrow failure or other affected organs. Idiopathic pulmonary fibrosis was diagnosed in one mutation-carrier individual. No other investigated illnesses tracked with mutational status.

## Results

### Family A

**Figure 1:**
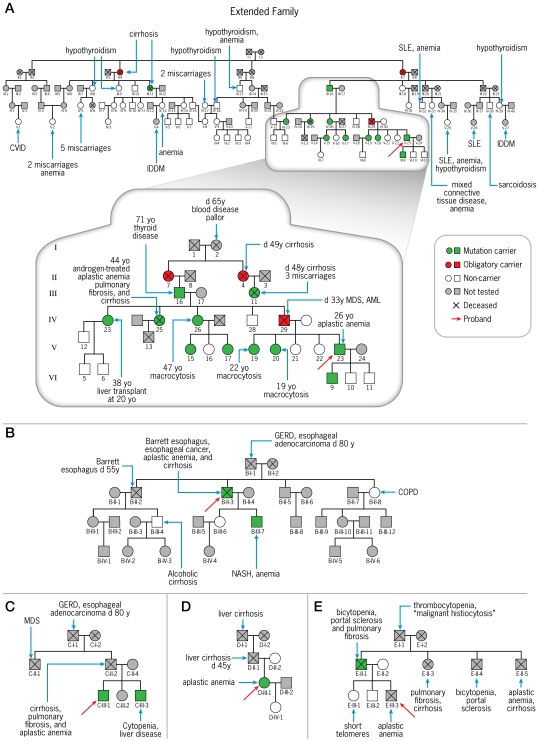
Pedigrees showing Telomerase Mutations and Disease Phenotypes. **(A)** The *TERT* K570N mutation tracked with hematological disorders and severe liver disease (lower pedigree) in Family A. In the extended family (upper pedigree), several disorders are found, including autoimmune diseases, anemia, thyroid diseases, liver diseases, and multiple miscarriages; however, the mutation was only associated with liver disease and multiple miscarriages. Two consanguineous relationships are not show: Subject A-IV-17 is a grand-daughter of Subjects A-II-7 and A-II-8, and Subject A-IV-7 is a grandson of Subjects A-III-14 and A-III-15. The genetic status for the immediate family (lower pedigree) and its association with bone marrow failure have been previously reported by us [15]. In smaller pedigrees, **(B)** *TERC* nucleotide 341-360 deletion tracked to liver disease in family B, **(C)** liver disease occurred in a family with a *TERC* nucleotide 28–34 deletion, and **(D)** in a family with *TERC* nucleotide 109–123 deletion. The following are denoted by their abbreviations: common variable immunodeficiency (CVID), aplastic anemia (AA), myelodysplastic syndrome (MDS), acute myeloid leukemia (AML), insulin-dependent diabetes mellitus (IDDM), systemic lupus erythematosus (SLE), idiopathic thrombocytic purpura (ITP), and non-alcoholic steatohepatitis (NASH).

**Figure 2:**
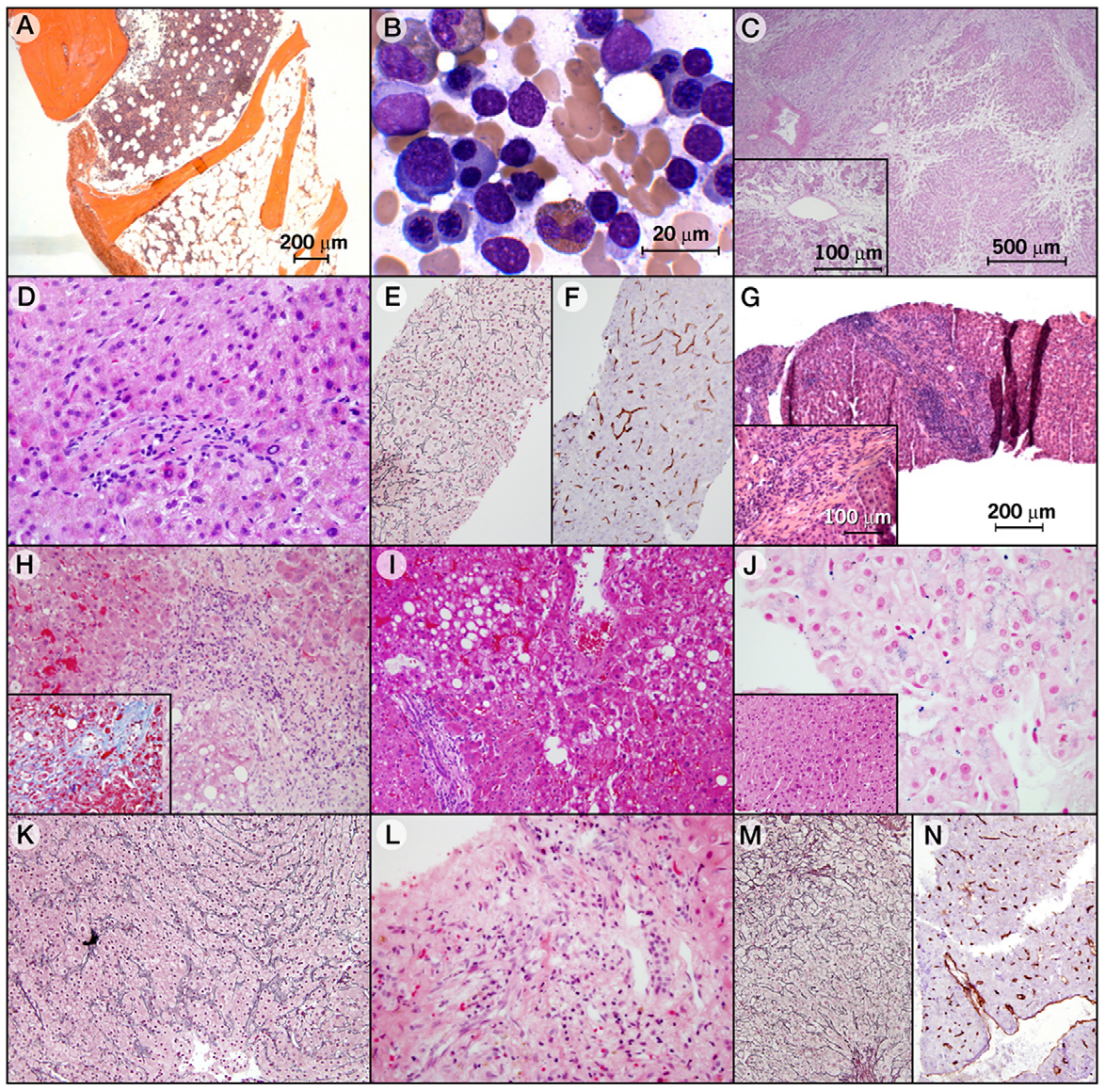
Hematoxylin and Eosin Bone Marrow and Liver Sections from Probands and Relatives with Aplastic Anemia, Acute Myeloid Leukemia, and Severe Liver Disease. **(A)** Family A proband's bone marrow was hypocellular with isolated regions of normal cellularity (hematoxylin and eosin [H&E] staining; low power magnification). **(B)** Proband's father's bone marrow smear (Subject A-IV-29), illustrating dysplastic changes and increased number of blasts (H&E, high power magnification). **(C)** Subject A-IV-23's liver biopsy revealing islands of liver surrounded by zones of necrosis and parenchymal collapse (H&E, low magnification). The necrosis was far enough in the past that hepatocytes have mostly disappeared. In the inset, in some of the areas where hepatocytes were preserved there was still necrosis near the central veins. Little evidence of inflammation exists. **(D)** Subject's A-IV-25's liver biopsy showing small portal areas and poorly formed veins (H&E, low power magnification). **(E)** Same liver biopsy exhibiting widened hepatocyte plates on the reticulin stain (high power magnification), but clear changes of nodular regenerative hyperplasia were not seen. **(F)** The CD34 stain by was positive in sinusoidal endothelial cells consistent with an abnormal proportion of arterial blood flow to the sinuses (immunohistochemistry, low power magnification). **(G)** Liver biopsy of Subject A-III-11 in which the hepatic architecture is distorted by bridging fibrosis (low power magnification); the inset gives a close-up of the fibrosis. The biopsy revealed moderate inflammation but not elevated levels of plasma cells relative to other inflammatory cells. Other changes included interface hepatitis and cholatestasis. **(H)** Subject B-II-3's liver biopsy demonstrating portal inflammation with interface hepatitis (H&E, low power magnification). In the inset, Masson staining shows sclerosis around central vein with perisinusoidal fibrosis. **(I)** Subject B-III-7's liver biopsy with mild, macrovesicular steatosis in a zone 3 distribution. The inset indicates that there is mild lymphocytic portal inflammation with focal interface hepatitis (H&E). **(J)** Subject C-III-3's liver biopsy shows mild hepatocellular iron accumulation in a pericanalicular pattern; the sinusoidal-lining cells show mild to moderate iron accumulation. The inset illustrates mild variation in hepatocyte nuclear size. **(K)** Subject C-III-3's reticulin staining exemplifying several zones in which the hepatocyte plates were abnormally widened, consistent with regeneration. **(L)** Subject E-II-1's liver biopsy revealing some portal areas with mild inflammation and all with poorly formed, slit-like veins (H&E). **(M)** The reticulin stain showed evidence of nodular regenerative hyperplasia, with zones of plate widening alternating with areas of compression. **(N)** CD34 stain was abnormally positive in the sinusoidal endothelial cells by immunohistochemistry, indicating abnormal proportion of arterial blood flow to the sinuses.

The proband (Subject A-V-23; **Fig. 1A**) presented at age twenty-five with a ten-year history of progressive pancytopenia (**Fig. 2A**). We have previously found that he was heterozygous for a loss-of-function *TERT* K570N mutation [15]. Eighteen members of his immediate family (**Fig. 1A**, inset) were previously screened and eight tested positive for the mutation [15]. A long history of hematologic diseases was well known in the family back to the patient's paternal great-great-grandmother, Subject A-I-2, who died of an apparent blood disorder at the age of sixty-five. However, the great-grandmother and the grandfather, Subjects A-II-7 and A-III-16 (the latter previously found to be positive for the mutation [15]), had not manifested hematologic symptoms. The proband's father (Subject A-IV-29) had thrombocytopenia and mild anemia from childhood. By age thirty-three, he developed myelodysplasia, which rapidly progressed to acute myeloid leukemia and death subsequent to an induction cycle of chemotherapy (**Fig. 2B**). The father was an obligatory carrier, as three of his sisters and his father also tested positive and his wife (the proband's mother) tested normal. Another of the proband's heterozygous paternal aunts (Subject A-IV-26) and the index patient's two heterozygous sisters, Subjects A-V-19 and A-V-20, ages forty-seven, twenty-two, and nineteen, respectively, only have macrocytosis, whereas his two wild-type sisters (Subjects A-V-21 and A-V-22) are healthy and without hematologic abnormalities. The patient's eldest of three sons, now six years old, carries the mutation, but he is asymptomatic and has normal blood counts.

The family has a history of severe liver disease. In our first report [15], only one paternal aunt (Subject A-IV-23) was found to carry the mutation and have liver disease. She underwent successful liver transplantation at age twenty for a non-A, non-B hepatitis that rapidly evolved to submassive hepatic necrosis with early fibrosis (**Fig. 2C**). Pathological examination of her liver uncovered massive necrosis without significant hepatitis, which is not specific for a particular etiology. Masson stain revealed early fibrosis in areas of parenchymal collapse and at the edges of portal areas and around central veins. She experienced anemia during pregnancy, but she otherwise had no history of hematologic abnormalities. The paternal aunt with a twenty-year history of aplastic anemia (Subject A-IV-25) and also heterozygous for the mutation developed dyspnea and cough at the age of forty-six after our first report. Spirometry revealed a moderately restrictive pattern and a very severe diffusion defect. Chest computed tomography showed heterogeneous bilateral peripheral lower lung infiltrates, suggestive of pulmonary fibrosis. Prednisone therapy was initiated, but she developed rapidly accumulating ascites, and was diagnosed with non-cirrhotic portal hypertension. She tested negative for hepatitis-associated viruses. Hepatocellular and canalicular enzymes were in the normal range, but her albumin was mildly low (3.6 g/dL) and total bilirubin was mildly increased (1.6 g/dL). Liver biopsy revealed no fibrosis connecting portal areas or perisinusoidal fibrosis. However, portal areas were small without visible vein (**Fig. 2D**). Hepatocytes showed variation in cell and nuclear size, and varying plate width, consistent with regeneration on reticulin stain (**Fig. 2E**). CD34 abnormally stained in the sinusoidal endothelial cells around the portal areas and central veins, consistent with an abnormal proportion of arterial blood flow to the sinuses (**Fig. 2F**). Iron was heavily accumulated, mainly in hepatocytes in zone 1. Her pulmonary function rapidly deteriorated, and she died of respiratory insufficiency.

We now expanded the genetic screening to an additional 35 relatives (**Fig. 1A**). Only one relative tested positive for the mutation, which was a first-cousin-twice-removed (Subject A-III-11) who died at the age of forty-eight with the diagnosis of liver cirrhosis. Her mutational status was demonstrated by sequencing DNA extracted from a paraffin-embedded liver biopsy obtained from hospital archives. She presented with pyoderma gangrenosum and upon workup, her liver enzymes and bilirubin were elevated (AST, 124 IU/L; alkaline phosphatase, 410 IU/L; albumin, 3.8 g/dL; total bilirubin, 3.8 mg/dL), and liver biopsy (**Fig. 2G**) demonstrated pre-cirrhotic chronic cholestatic liver disease with a differential diagnosis of primary sclerosing cholangitis or a sclerosing cholangitis secondary to autoimmune hepatitis. Serology for hepatitis A and B were negative at the time. She was treated with azathioprine and prednisone for one month, after which time she became severely jaundiced. Her clinical state deteriorated, and she died of fungemia. Her mother, a great-great-aunt to the index patient (Subject A-II-4) died at forty-nine of liver cirrhosis. Unfortunately, no biopsy specimen or clinical records were available. Because her husband was not related to the proband, and her daughter (A-III-11) carried the mutation, she was an obligatory carrier (**Fig. 1A**).

There was no nail dystrophy, leukoplakia, or skin hyperpigmentation, all physical features characteristic of dyskeratosis congenita, in any of the mutation-carriers. Although the index patient and some of his relatives showed a strikingly premature graying of hair, surprisingly this characteristic did not track with the mutation. No history of alcohol consumption or smoking was present in the family.

### Family B

The proband (Subject B-II-3; **Fig 1B**), a 57-year-old man heterozygous for a novel *TERC* nucleotide 341–360 deletion not found in 188 controls, presented a six-year history of Barrett's esophagus and recently worsening dysphagia. Imaging displayed a tumor at the gastro-esophageal junction, which was identified as esophageal cancer by biopsy. During the initial evaluation, the patient was pancytopenic, and his bone marrow revealed 5% cellularity with trilineage hypoplasia but without malignant infiltration. His liver enzymes and function tests also were abnormal (alkaline phosphatase, 482 IU/L; albumin, 2.2 g/dL; total bilirubin, 0.9 mg/dL). The tumor was surgically resected, and concurrent liver biopsy showed cirrhosis with foci of lobular inflammation dominated by plasma cells, extensive sinusoidal fibrosis, and Mallory bodies (**Fig. 2H**). Serological tests for hepatitis-associated viruses were negative. His father (Subject B-I-1) had suffered from gastroesophageal reflux disease and died at age eighty of a poorly differentiated adenocarcinoma at the gastroesophageal junction with hepatic metastasis. The proband's brother (Subject B-II-2) also had gastroesophageal reflux disease and Barrett's esophagus. No pathological specimens were available from his father and his brother for genetic screening. His brother's son (Subject B-III-4) was reported to abuse alcohol and to have cirrhosis, but he tested negative for the mutation. The proband's thirty-two year-old son (Subject B-III-7) is heterozygous for the *TERC* deletion; he had mild macrocytic anemia during a routine medical visit. Upon further investigation, he was found to have an enlarged fatty liver on ultrasound though his liver enzymes were normal; his bone marrow biopsy was hypocellular (20%), and his liver biopsy showed macrovesicular steatosis with foci of lobular inflammation, portal chronic inflammatory infiltrate, and mild hepatocellular iron accumulation (**Fig. 2I**). Tests for hepatitis B and hepatitis C viruses were all negative. Pulmonary function test results were within normal limits. He has an eleven-year history of social alcohol consumption.

### Family C

The proband (Subject C-III-1; **Fig. 1C**), a thirty year-old Caucasian male previously fond to be heterozygous for a *TERC* nucleotide 28-34 deletion [15], had a thirteen-year history of moderate aplastic anemia. His father (Subject C-II-2) had a long history of thrombocytopenia and died at age thirty-two of fungal sepsis; autopsy revealed mixed micro and macronodular liver cirrhosis, chronic congestive splenomegaly, esophageal varices, diffuse interstitial pulmonary fibrosis with mild chronic inflammation, and a mildly hypoplastic bone marrow (50%). The index patient's paternal uncle (Subject C-II-1) had died of myelodysplasia. We now screened his brother (Subject C-III-3), with a long history of mild pancytopenia and elevated liver enzymes, and he also tested positive for the *TERC* deletion. Liver biopsy demonstrated hepatocytes with mild variation in nuclear size, mild hepatocellular iron accumulation in a pericanalicular pattern (**Fig. 2J**), and several zones displaying abnormally widened hepatocyte plates, consistent with regeneration (**Fig. 2K**).

### Family D

The proband, a thirty-eight year-old female, was found to be heterozygous for a novel *TERC* nucleotide 109–123 deletion not observed in 188 controls. She had a six-year history of transfusion-independent pancytopenia first detected during pregnancy. At that time, her hemoglobin was 6 g/dL, and her anemia was unresponsive to erythropoietin treatment. Her bone marrow biopsy was 5% cellular with trilineage hypoplasia and a transient clonal chromosome 1 abnormality [der(1) t(1;1)(p36.2;q;12)]. Her wild-type mother (Subject D-II-2) had history of resolved anemia in the past but is otherwise healthy. Her father (Subject D-II-1), a probable carrier as her mother tested negative, had a 15-year history of hepatitis and liver cirrhosis and died at the age of forty-five years of massive gastrointestinal bleeding. At necropsy, the liver was cirrhotic and microscopically showed moderate fatty change and hyaline Mallory bodies; spider angiomata, jaundice, ascites, and esophageal varices also were present. He had a history of moderate alcohol consumption. Unfortunately, no pathologic specimen was available for further analysis or genetic testing. The index patient's paternal grandfather, (Subject D-I-1) also died of cirrhosis at a young age (pathologic specimens were not available).

### Family E

The proband (Subject E-III-3), a fifteen-year-old male, presented in 1992 with a history of hemorrhage. Laboratory tests revealed pancytopenia and elevated alkaline phosphatase; liver function tests were within normal limits. Bone marrow biopsy showed 25% cellularity with normal cytogenetics. He was treated with androgens without much benefit for his blood counts. As his hematological status deteriorated, he underwent an unrelated hematopoietic stem cell transplant but died of a transplant-related complication. His father (Subject E-II-1) was forty-four years old when first seen, and at the time, he had a 10-year history of thrombocytopenia and leukopenia along with a mildly hypocellular bone marrow. This unusual association between aplastic anemia and liver cirrhotic disease observed in this pedigree and in an additional family led us to describe a “new familial syndrome” in 1997, which appeared to have an autosomal dominant inheritance, but genetic analysis was not available at that time (family E in the present series corresponds to family A in our previous report) [16]. Six years post-presentation, the father developed a nonproductive cough and dyspnea on exertion. Spirometry revealed a reduced diffusion capacity and computed tomography of the chest was consistent with pulmonary fibrosis. During evaluation, some liver enzymes were elevated, and liver biopsy findings were consistent with hepatoportal sclerosis complicated by nodular regenerative hyperplasia. He had no history of ethanol consumption or smoking. Microscopic examination revealed portal areas with chronic inflammatory infiltrate but with no interface hepatitis (**Fig. 2L**). The portal veins were either missing or slit-like in most of the portal areas. Hepatic architecture was subtly distorted by nodularity with zones of small compressed hepatocytes alternating with zones of large hepatocytes with widened plates (**Fig. 2M**). CD34 staining was abnormally positive in sinusoidal endothelial cells, mainly around the portal areas and central veins (**Fig. 2N**). There was sinusoidal dilatation and congestion near central veins. Iron was accumulated within hepatocytes in zones one and two. Ultimately, the respiratory symptoms evolved, and the patient died of respiratory insufficiency. Serological tests for hepatitis B and hepatitis C viruses were negative. Sixteen years post-presentation, the DNA extracted from his paraffin-embedded liver specimen revealed a novel heterozygous *TERT* S368F mutation not present in 528 healthy controls. The proband's paternal grandfather (Subject E-I-1) died at age sixty-four, and autopsy revealed pulmonary fibrosis, nodular hyperplasia of the liver, and splenomegaly. A paternal aunt (Subject E-II-3) died at age thirty-seven with extensive pulmonary fibrosis and liver cirrhosis. She presented macrocytosis in peripheral blood but normocellular bone marrow. A paternal uncle (Subject E-II-4) died at age twenty-three with massive gastrointestinal bleeding and thrombocytopenia. Autopsy revealed splenomegaly with expanded red pulp, consistent with portal hypertension, and the liver appearance was consistent with portal fibrosis but not cirrhosis. The other paternal uncle (Subject E-II-5) died at age thirty-five years, and autopsy revealed aplastic anemia, splenomegaly, macronodular cirrhosis, and portal fibrosis. No samples were available for the genetic testing of the other affected individuals, including the proband. The proband's two siblings (Subjects E-III-1 and E-III-2), mother (Subject E-II-2), and cousin (Subject E-III-4) appear to be healthy, and each tested negative for the mutation.

Further details of the clinical cases described here are available to other researchers on request.

### Leukocyte Telomere Length and Genotype

**Figure 3:**
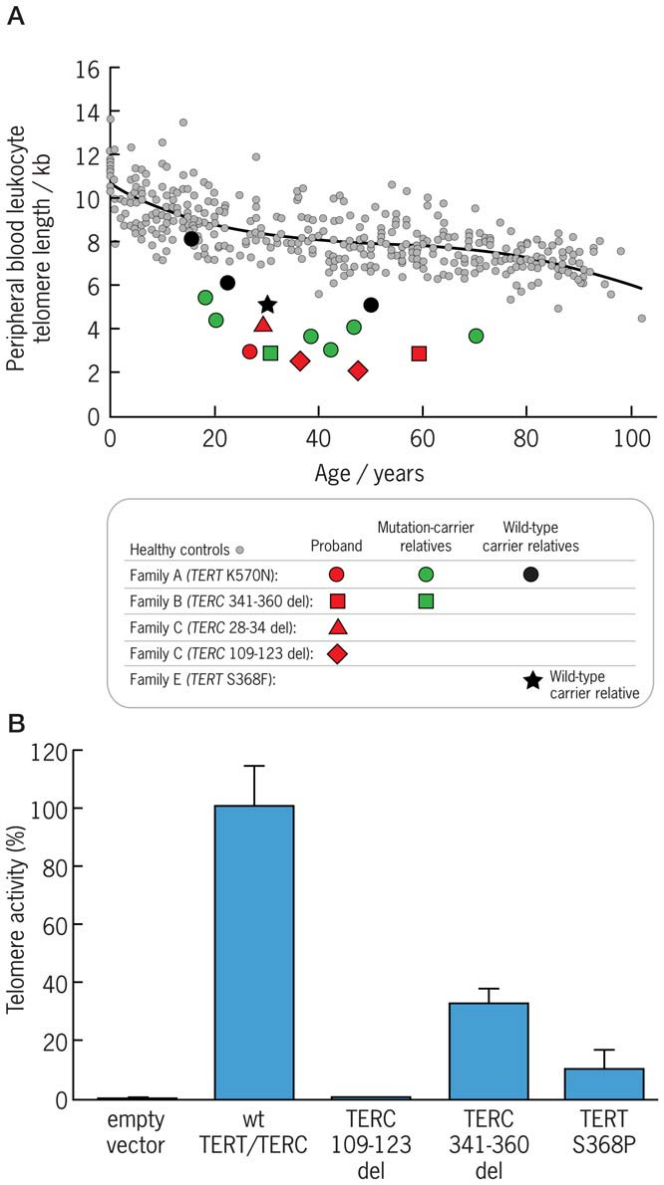
Functional analysis of telomerase mutations. **(A)** Telomere length in peripheral-blood total white blood cells (ordinate) from patients and their relatives with or without telomerase gene mutations as a function of age (abscissa) compared to healthy controls. Telomere lengths were measured by flow fluorescence *in situ* hybridization (flow-FISH). Small gray circles represent the telomere lengths for 400 healthy volunteers [10], and the curve marks the 50^th^ percentile for healthy controls as a function of age. **(B)** Telomerase activity - measured by telomeric-repeat amplification assay - of lysates of telomerase-negative WI38-VA13 cells cotransfected with mutated *TERC* and wild-type *TERT* expression vectors (2 µg per vector per transfection reaction). Enzymatic activity was normalized to *TERC* expression as measured by Real Time RT-PCR and to the telomerase activity of wild-type *TERC*, which was set at 100%. Quadruplicate measurements were performed using one microgram of cell lysate protein per reaction. “Empty vector” refers to protein from VA13 cells transfected with an empty pcDNA3-Flag vector in lieu of *TERC*.

In the three generations of Family A analyzed by flow-FISH, mutation carriers (Subjects A-III-16, A-IV-23, A-IV-25, A-IV-26, A-V-19, A-V-20, and A-V-23) had total peripheral blood white cell telomere lengths below the shortest percentile of healthy age-matched controls (**Fig. 3A**). In contrast, the wild-type individuals analyzed (Subjects A-IV-28, A-V-21, and A-V-22) were found to have telomere lengths between those of their heterozygous family members and the 50th percentile. In the additional four families studied, all tested mutation carriers had lymphocyte and neutrophil telomere length below the shortest percentile (**Fig. 3A**). Interestingly, four non-carriers in families A and E, each of whom had a heterozygous parent, also had short telomeres for their age (**Fig. 3A**).

### Mutation Functional Analysis

We previously showed that *TERT* K570N results in a complete loss of the ability of telomerase to add hexameric repeats to telomeres and that *TERC* nucleotide 28–34 deletion reduces telomerase enzymatic activity - each by haploinsufficiency [15]. Here we found by co-transfection experiments that *TERC* nucleotide 109–123 deletion completely abolished telomerase enzymatic activity, *TERC* nucleotide 341–360 deletion reduced telomerase activity to approximately one-third of that observed for wild-type *TERC*, and *TERT* S368P reduced telomerase activity to approximately 10% of that observed for wild-type *TERT* (**Fig. 3B**).

## Discussion

Hepatic disease is mentioned in reviews of dyskeratosis congenita, estimated at about seven percent of patients, but not well characterized and often blamed on hemochromatosis from frequent blood transfusions [14]. A few case reports describe cirrhosis and hepatic cell necrosis in affected individuals in autosomal dominant pedigrees [8], [11], [17]. Liver complications are described as more frequent and severe in occasional case reports of bone marrow transplantation in dyskeratosis congenita [18]. In a recent series of 150 patients with idiopathic interstitial pneumonias, four patients (3%) also had cryptogenic liver cirrhosis diagnosed in the sixth or seventh decades of life; none of these four patients, however, carried a telomerase mutation [17].

Our families did not present in childhood or display the characteristic physical anomalies typical of dyskeratosis congenita, except for the premature graying of hair in families A and C that did not track with the mutations. Similar to the spectrum of hematological findings associated with telomerase mutations, ranging from isolated macrocytosis to acute myeloid leukemia, liver disease was heterogeneous in severity and pathology among telomerase-mutation carriers. However, in our comprehensive histopathological analysis, some findings were recurrent: most patients had both inflammatory and fibrotic components; several patients developed cirrhosis; individuals from three different families (A, C, and E) had histological findings consistent with hepatic nodular regeneration (Table 1). In others, iron accumulation was observed, in the absence of a history of blood transfusion or *HFE* gene mutation. In two instances from different families, CD34 stained positive in sinusoidal endothelial cells, consistent with portal hypertension. Alcohol consumption was observed in affected individuals in families B and D, suggesting a role for environmental factors triggering organ injury; however, serologies for viral hepatitis were negative for all individuals tested. Of interest, more than a decade ago, we identified two families with a “new familial syndrome” characterized by a combination of bone marrow failure and chronic liver disease [16]. A recent study reports a family with pulmonary fibrosis, hepatic nodular regenerative hyperplasia, and aplastic anemia [19] and another describes a case of regenerative hyperplasia and aplastic anemia [20]. Unfortunately, telomerase complex genes were not sequenced in these two families. The wide range in clinical phenotypes associated with telomerase mutations is compatible with the variable genetic penetrance of these mutations and of their effects on telomere shortening. The variety in histopathological findings in liver specimens also suggests that other genetic, epigenetic, and environmental factors are essential for disease development and progression.

**Table 1:** Hepatic profile of patients with telomerase mutations and liver disease.

Subject	Age/Gender (years)	Liver function tests	Liver histology
A-IV-23	20/F	(not available)	Massive necrosis without significant hepatitis; some early fibrosis in areas of parechymal collapse as well as at the edges of portal areas and around central veins
A-IV-25	46/F	Bilirubin mildly elevated; albumin mildly decreased	Hepatocyte variation in cell and nuclear size, and variation in plate width, consistent with regeneration on reticulin stain; CD34 stained positive in sinusoidal endothelial cells; iron heavily accumulated, mainly in hepatocytes in zone 1
A-III-11	48/F	Enzymes elevated; normal albumin	Hepatic architecture is distorted by bridging fibrosis; moderate inflammation, hepatitis, and cholatestasis
B-II-3	57/M	Alkaline phosphatase elevated; normal bilirubin; low albumin	Cirrhosis with foci of lobular inflammation dominated by plasma cells, extensive sinusoidal fibrosis, and Mallory bodies
B-III-7	32/M	normal	Macrovesicular steatosis with foci of lobular inflammation and portal chronic inflammatory infiltrate and mild hepatocellular iron accumulation
C-III-3	30/M	Enzymes elevated	Hepatocytes with mild variation in nuclear size, mild hepatocellular iron accumulation in a pericanalicular pattern, and several zones displaying abnormally widened hepatocyte plates
E-II-1	52/M	Enzymes elevated	Nodular regenerative hyperplasia with zones of small compressed hepatocytes alternating with zones of large hepatocytes with widened plates; CD34 positive in sinusoidal endothelial cells mainly around the portal areas and central veins

Our current findings parallel the recently reported association of loss-of-function *TERT* and *TERC* mutations with familial idiopathic pulmonary fibrosis [12], [13]. As was hypothesized for pulmonary fibrosis, shortened telomeres may result from dysfunctional telomere repair, increased cell turn-over, or a combination of factors and contribute to liver fibrosis.

In murine models, chronic chemical liver injury is associated with increased regeneration defects and liver cirrhosis in telomerase-deficient mice; restoration of telomerase activity by gene transduction abrogates liver cirrhosis and improves liver function [21]. Short and dysfunctional telomeres in *Tert*-deficient (and p53-mutated) mice also increase susceptibility to toxin-induced hepatocellular carcinoma [22]. In murine livers lacking telomeric repeat binding factor 2, hepatocytes remained viable and regenerated despite telomeric deprotection and fusion through endoreduplication [23]. Telomeres in liver cells might be maintained by recombination only as long as lengthy telomere repeat tracts are available on some chromosome ends, which is unlikely when telomerase is deficient. Alternatively, the presence of inflammatory cells in liver sections even at very early stages of liver disease (Subject B-III-7, **Fig. 2I**) suggests that these cells may be the key mediators of pathogenic fibrosis in the setting of telomere shortening. At damaged sites of chronically injured tissues or organs, such as the liver and lung, the release of inflammatory mediators recruits leukocytes to the extracellular matrix [24]. T cells chronically secrete profibrotic cytokines that activate macrophages and fibroblasts, which subsequently stimulate myofibroblasts, which may be of bone marrow derivation. Telomere erosion in neutrophils and lymphocytes, cells critical to the inflammatory response, may elicit an abnormal, sustained profibrotic response.

The pathophysiology for hepatic nodular regenerative hyperplasia is unknown. It is associated with vasculitis and exposure to drugs, such as azathioprine; one case in our series (Subject A-III-11) developed fatal liver disease after azathioprine administration. More than five percent of autopsied individuals over eighty years old have nodular regenerative hyperplasia, and portal hypertension is a major complication [25]. Taken together, the link of telomerase deficiency to pulmonary fibrosis and the present findings open a new perspective in the investigation of inflammation, regeneration, and fibrosis and lend support to a crucial role of telomerase in these processes. Up-regulation of telomerase expression and activity may be an attractive therapeutic target for the treatment of fibrotic and regenerative diseases.

In family A, both the patient and his father showed clonal evolution of a malignant cell population, a finding also observed in families C and D. Leukemic cells appear to require telomerase activity for proliferation. However, telomerase deficiency increases the presence of critically short telomeres, which are prone to chromosomal instability. Myelodysplasia and acute leukemia are observed in classic dyskeratosis congenita in 1% [26] to 3% of cases [14], and the risk of developing acute myeloid leukemia in dyskeratosis congenita patients is increased almost 200 fold in comparison to the expected incidence in the population [27]. We recently found an increased rate of constitutional *TERT* hypomorphic mutations in patients with acute myeloid leukemia [28]. Telomerase mutations were associated with cytogenetic abnormalities, especially trisomy 8 and inv(16). Short telomeres may limit normal stem cell division by inducing proliferation arrest and select for stem cells with dysfunctional telomeres and defective DNA damage responses that are prone to chromosomal instability. Additionally, genome-wide association studies implicated *TERT* as a strong susceptibility locus (chr 5p15.33) for a large variety of cancers [29]–[32].

Notable characteristics of telomerase mutations in these families include incomplete penetrance and variable expressivity. Some family members have the mutation and short telomeres but appear healthy. Of clinical relevance, these findings indicate that the presence of a telomerase gene mutation and very short telomeres do not necessarily translate into disease. In addition, the phenotypes associated with the mutation are quite disparate in nature. Mutations in telomerase and short telomeres must work in concert with other genetic and environmental factors to result in the diverse phenotypes with which these mutations now have been associated.

## Methods

### Ethics Statement

Patients and their relatives or guardians of minors provided written informed consent for genetic testing, according to protocols approved by the institutional review board of the National Heart, Lung, and Blood Institute, protocol 04-H-0012 (www.ClinicalTrials.gov identifier: NCT00071045). Clinical investigation was conducted according to the principles expressed in the Declaration of Helsinki. Continuing review application of the protocol was last renewed by NHLBI IRB in February 26^th^, 2009; the protocol was last amended on September 9^th^, 2009. Patients who are described in detail have read the manuscript and provided written informed consent for publication.

### Patients and Controls

The probands of each family were referred to the National Institutes of Health Hematology Branch clinic for evaluation of bone marrow failure, and they were diagnosed with aplastic anemia based on conventional bone marrow and blood-count criteria [33]. In Family A, relatives were invited for clinical and genetic evaluation and responded a questionnaire regarding their health status and specifically addressing hematologic, immune, pulmonary, and hepatic diseases. When a clinical history was positive, subjects were requested to provide clinical tests and medical records for further analysis. Medical records also were obtained from deceased relatives who had had liver or pulmonary disease, upon family approval. In the other families, relatives were invited for clinical evaluation at the NIH Clinical Center and medical records from outside institutions were obtained after consent; medical records also were obtained from deceased relatives, upon family consent. Complete blood counts were performed at the NIH Clinical Center for clinically healthy subjects found to carry a telomerase mutation, and the diagnosis of macrocytosis or anemia were established based on the standard laboratory parameters. Patients and their relatives or guardians of minors provided written informed consent for genetic testing, according to protocols approved by the institutional review board of the National Heart, Lung, and Blood Institute. DNA was extracted from peripheral-blood or buccal mucosal cells as previously described [34]; for Subject A-III-11 and Subject E-II-1, DNA was obtained from paraffin-embedded liver tissue (PicoPure DNA Extraction Kit, Arcturus, Mt View, CA).

DNA samples from 188 healthy persons served as controls for *TERC* and *TERT* gene mutations: 117 were white (94 from Human Variation Panel HD100CAU, Coriell Cell Repositories [http://locus.umdnj.edu/nigms/cells/humdiv.html], and 23 from SNP500Cancer [http://snp500cancer.nci.nih.gov]), 24 black (from SNP500Cancer), 23 Hispanic (from SNP500Cancer), and 24 Asian (from SNP500Cancer) [35]. An additional 340 healthy controls were screened for *TERT* gene mutations: 94 blacks from Human Variation Panel HD100AA and 246 anonymous healthy subjects of Hispanic origin (52 percent Peruvians, 28 percent Latin Americans, and 20 percent Pima and Maya Amerindians) [10].

### Mutational Analysis

*TERT* and *TERC* genotyping was performed as previously described [10].

### Telomere Length by Flow-FISH

Telomere length of peripheral blood leukocytes was measured after red cell lysis with ammonium chloride solution by flow cytometry-fluorescent *in situ* hybridization (flow-FISH) as reported previously [15], [36].

### Telomerase Enzymatic Activity

Wild-type vector was mutagenized by Mutagenex, and sequence was confirmed by direct sequencing of the whole insert, and plasmids were purified using the HiSpeed Plasmid Maxi Kit (Qiagen). Vectors containing disease-associated telomerase mutations were transfected into VA13 cells, and telomerase activity measured as previously described with slight modifications [10]. In the present study, we used a fluorescent telomere repeat amplification protocol (TRAP), XL TRAPeze assay (Chemicon) and fluorescence was measured in a Victor 3 multilabel plate reader (Perkin Elmer). Telomerase activity was measured in total product generated (TPG) units based on the standard curve results and calculated strictly according to the manufacturer's manual and expressed as telomerase activity relative to wild-type *TERT* and *TERC*, which was considered 100%.
